# Supplementary material for: MetaRibo-Seq measures translation in microbiomes
Source: Nat Commun. 2020 Jun 29;11:3268. doi: 10.1038/s41467-020-17081-z (PMC7324362; doi:10.1038/s41467-020-17081-z)
Supplement: Supplementary file 10 — Supplementary Data 7 [file 41467_2020_17081_MOESM10_ESM.zip › File2/Confidence_High_Taxonomy/155214_out.krona.html]

Javascript must be enabled to view this page.

members
magnitude
magnitudeUnassigned
count
unassigned
taxon
rank

155214\_out

297

superkingdom
2
297

phylum
1239
2

class
186801
2

186802
order
2

2
family
186803

841
genus
1

species
2049040
1

SRS142503\_contig\_number\_3787

1
33042
genus


SRS012969\_contig\_number\_2102
1
species
1896998

phylum
976
295

295
class
200643

295
order
171549

294
family
815

genus
816
294
58

SRS013951\_contig\_number\_contig-100\_3972.234966SRS016095\_contig\_number\_contig-100\_1199.167866SRS016897\_contig\_number\_contig-100\_3723.25292SRS016989\_contig\_number\_15819SRS017247\_contig\_number\_contig-100\_4550.65854SRS017375\_contig\_number\_contig-100\_6458.6459SRS018817\_contig\_number\_contig-100\_2233.133230SRS019910\_contig\_number\_contig-100\_3578.120913SRS020508\_contig\_number\_contig-100\_1013.18751SRS021948\_contig\_number\_contig-100\_1879.39562SRS023346\_contig\_number\_6985SRS024388\_contig\_number\_contig-100\_4019.94121SRS024435\_contig\_number\_contig-100\_3123.233085SRS042628\_contig\_number\_31316SRS044535\_contig\_number\_22890SRS049773\_contig\_number\_contig-100\_8627.8628SRS049995\_contig\_number\_37774SRS050520\_contig\_number\_contig-100\_775.776SRS054905\_contig\_number\_contig-100\_8530.88783SRS056537\_contig\_number\_contig-100\_6672.48412SRS057717\_contig\_number\_5803SRS058268\_contig\_number\_contig-100\_970.971SRS063985\_contig\_number\_2178SRS064232\_contig\_number\_contig-100\_1176.39368SRS064757\_contig\_number\_8646SRS075773\_contig\_number\_contig-100\_26933.26934SRS076976\_contig\_number\_4668SRS077231\_contig\_number\_contig-100\_1395.156935SRS077849\_contig\_number\_30556SRS078176\_contig\_number\_contig-100\_6571.107189SRS097920\_contig\_number\_24642SRS098881\_contig\_number\_26238SRS1041032\_contig\_number\_contig-100\_416.67736SRS1041090\_contig\_number\_contig-100\_3985.57828SRS1041130\_contig\_number\_contig-100\_2856.52796SRS1041141\_contig\_number\_7180SRS1041143\_contig\_number\_contig-100\_8791.38583SRS1041164\_contig\_number\_6240SRS1054716\_contig\_number\_contig-100\_3355.3356SRS1054928\_contig\_number\_contig-100\_5416.47252SRS1055076\_contig\_number\_contig-100\_3967.102805SRS142923\_contig\_number\_contig-100\_20422.20423SRS143085\_contig\_number\_2206SRS143780\_contig\_number\_contig-100\_21582.55564SRS144714\_contig\_number\_22367SRS147139\_contig\_number\_contig-100\_7949.220591SRS147346\_contig\_number\_contig-100\_10667.298207SRS147653\_contig\_number\_contig-100\_1186.56517SRS147727\_contig\_number\_contig-100\_922.923SRS148253\_contig\_number\_contig-100\_7078.7079SRS148817\_contig\_number\_2621SRS148970\_contig\_number\_contig-100\_1554.30178SRS149784\_contig\_number\_contig-100\_6130.119816SRS150029\_contig\_number\_contig-100\_7896.52227SRS893172\_contig\_number\_contig-100\_17619.54206SRS893300\_contig\_number\_contig-100\_3989.83443SRS893327\_contig\_number\_12028SRS893373\_contig\_number\_contig-100\_11722.94115

species
457387
8

SRS016495\_contig\_number\_19823SRS043667\_contig\_number\_3733SRS053214\_contig\_number\_13928SRS053335\_contig\_number\_15654SRS075984\_contig\_number\_contig-100\_398.167012SRS104327\_contig\_number\_3878SRS147766\_contig\_number\_6798SRS893285\_contig\_number\_3852


SRS148424\_contig\_number\_contig-100\_1698.170289
1739517
species
1


SRS011405\_contig\_number\_17523SRS012849\_contig\_number\_33587SRS014287\_contig\_number\_17108SRS016753\_contig\_number\_7465SRS017521\_contig\_number\_51311SRS017859\_contig\_number\_7256SRS021153\_contig\_number\_3498SRS022524\_contig\_number\_14899SRS023971\_contig\_number\_559SRS024009\_contig\_number\_8433SRS024625\_contig\_number\_21026SRS042284\_contig\_number\_contig-100\_4568.46479SRS045244\_contig\_number\_4327SRS045645\_contig\_number\_8052SRS045739\_contig\_number\_8427SRS046502\_contig\_number\_9073SRS051610\_contig\_number\_425SRS052027\_contig\_number\_16572SRS062427\_contig\_number\_2893SRS075716\_contig\_number\_2511SRS076804\_contig\_number\_7237SRS077589\_contig\_number\_4641SRS101376\_contig\_number\_28811SRS1041118\_contig\_number\_4823SRS140492\_contig\_number\_34208SRS143148\_contig\_number\_1261SRS143895\_contig\_number\_11660SRS146813\_contig\_number\_12522SRS147377\_contig\_number\_8477SRS148784\_contig\_number\_29538SRS148874\_contig\_number\_16636SRS892882\_contig\_number\_6443SRS893279\_contig\_number\_11215SRS893380\_contig\_number\_1478SRS971275\_contig\_number\_32280
35
371601
species

species
820
3

SRS013800\_contig\_number\_contig-100\_1810.158129SRS017307\_contig\_number\_26397SRS145308\_contig\_number\_1346


SRS049402\_contig\_number\_10394SRS064973\_contig\_number\_1301SRS149879\_contig\_number\_26138
3
2292951
species


SRS013158\_contig\_number\_2384
1
species
46506


SRS077730\_contig\_number\_1855
817
species
1

8
338188
species

SRS013098\_contig\_number\_49736SRS015663\_contig\_number\_37198SRS017821\_contig\_number\_28603SRS018133\_contig\_number\_contig-100\_3312.77699SRS019397\_contig\_number\_11568SRS019685\_contig\_number\_35889SRS020622\_contig\_number\_6191SRS023583\_contig\_number\_16180


SRS013940\_contig\_number\_5712SRS014855\_contig\_number\_contig-100\_1229.106161SRS018351\_contig\_number\_15912SRS018936\_contig\_number\_12368SRS024132\_contig\_number\_29871SRS075078\_contig\_number\_13267SRS078419\_contig\_number\_32954SRS1041138\_contig\_number\_10982SRS142980\_contig\_number\_7219SRS148721\_contig\_number\_55051SRS893170\_contig\_number\_contig-100\_2781.70674
species
585544
11


SRS075878\_contig\_number\_210SRS893256\_contig\_number\_412
2
species
818

species
2292957
8

SRS011239\_contig\_number\_12013SRS014736\_contig\_number\_contig-100\_1191.69807SRS018427\_contig\_number\_contig-100\_1006.187170SRS019178\_contig\_number\_contig-100\_2768.2769SRS1041039\_contig\_number\_contig-100\_2583.158614SRS1041146\_contig\_number\_contig-100\_4335.4336SRS1055067\_contig\_number\_contig-100\_3812.90367SRS1055099\_contig\_number\_contig-100\_1941.92163

1
species
2292938

SRS147977\_contig\_number\_4455


SRS011084\_contig\_number\_31941SRS011302\_contig\_number\_27484SRS012273\_contig\_number\_44262SRS012902\_contig\_number\_contig-100\_717.17945SRS013215\_contig\_number\_2341SRS013476\_contig\_number\_26002SRS013521\_contig\_number\_10405SRS013687\_contig\_number\_33789SRS014235\_contig\_number\_47643SRS014459\_contig\_number\_17337SRS014683\_contig\_number\_1776SRS014979\_contig\_number\_36303SRS015065\_contig\_number\_32019SRS015264\_contig\_number\_22389SRS015369\_contig\_number\_6897SRS015431\_contig\_number\_87180SRS015579\_contig\_number\_23403SRS015694\_contig\_number\_8189SRS015794\_contig\_number\_13555SRS015854\_contig\_number\_3099SRS015960\_contig\_number\_18071SRS016018\_contig\_number\_3959SRS016203\_contig\_number\_10089SRS016840\_contig\_number\_2857SRS016954\_contig\_number\_26573SRS017103\_contig\_number\_43673SRS017191\_contig\_number\_14134SRS017433\_contig\_number\_contig-100\_4569.117977SRS017622\_contig\_number\_121SRS018313\_contig\_number\_2647SRS018888\_contig\_number\_contig-100\_318.56703SRS019030\_contig\_number\_1492SRS019068\_contig\_number\_117853SRS019381\_contig\_number\_11059SRS019601\_contig\_number\_29745SRS019693\_contig\_number\_1963SRS019968\_contig\_number\_15984SRS020271\_contig\_number\_4501SRS020869\_contig\_number\_12548SRS021219\_contig\_number\_13138SRS021484\_contig\_number\_28021SRS022071\_contig\_number\_37552SRS022609\_contig\_number\_51642SRS022713\_contig\_number\_4737SRS023526\_contig\_number\_37795SRS023715\_contig\_number\_24SRS023829\_contig\_number\_contig-100\_1403.13167SRS024331\_contig\_number\_26223SRS024492\_contig\_number\_2358SRS024549\_contig\_number\_contig-100\_1929.43335SRS024663\_contig\_number\_11594SRS043001\_contig\_number\_7731SRS043701\_contig\_number\_2687SRS045004\_contig\_number\_16904SRS045195\_contig\_number\_7978SRS046369\_contig\_number\_16635SRS046712\_contig\_number\_3518SRS046717\_contig\_number\_7904SRS047014\_contig\_number\_10091SRS048164\_contig\_number\_31627SRS049446\_contig\_number\_14307SRS049959\_contig\_number\_7404SRS050026\_contig\_number\_86SRS050299\_contig\_number\_13130SRS050941\_contig\_number\_contig-100\_409.145865SRS052576\_contig\_number\_686SRS052697\_contig\_number\_34372SRS053398\_contig\_number\_22831SRS053573\_contig\_number\_505SRS054352\_contig\_number\_contig-100\_157.138941SRS056259\_contig\_number\_20895SRS056273\_contig\_number\_30976SRS057478\_contig\_number\_5730SRS057480\_contig\_number\_contig-100\_773.68875SRS062654\_contig\_number\_28399SRS063040\_contig\_number\_47912SRS063518\_contig\_number\_32428SRS063928\_contig\_number\_contig-100\_3285.3286SRS064557\_contig\_number\_20994SRS065504\_contig\_number\_23429SRS074964\_contig\_number\_5478SRS075398\_contig\_number\_contig-100\_617.26569SRS076756\_contig\_number\_contig-100\_1223.135247SRS076929\_contig\_number\_contig-100\_12399.12399SRS077024\_contig\_number\_12917SRS077086\_contig\_number\_8196SRS077194\_contig\_number\_32566SRS077502\_contig\_number\_contig-100\_1681.100679SRS078242\_contig\_number\_29404SRS078665\_contig\_number\_760SRS097958\_contig\_number\_24047SRS098061\_contig\_number\_12047SRS098073\_contig\_number\_15009SRS098644\_contig\_number\_contig-100\_2053.258263SRS098655\_contig\_number\_12380SRS103987\_contig\_number\_contig-100\_1426.210030SRS104084\_contig\_number\_4890SRS1041036\_contig\_number\_2248SRS1041037\_contig\_number\_11277SRS1041038\_contig\_number\_9436SRS1041092\_contig\_number\_834SRS1041134\_contig\_number\_8679SRS1041136\_contig\_number\_26904SRS1041137\_contig\_number\_32309SRS1041144\_contig\_number\_8256SRS1041145\_contig\_number\_37823SRS1041147\_contig\_number\_1642SRS104311\_contig\_number\_43481SRS104400\_contig\_number\_50090SRS104975\_contig\_number\_25103SRS105153\_contig\_number\_9934SRS1055034\_contig\_number\_11944SRS1055043\_contig\_number\_15446SRS1055056\_contig\_number\_13972SRS1055083\_contig\_number\_13470SRS1055095\_contig\_number\_607SRS140513\_contig\_number\_4833SRS140645\_contig\_number\_328SRS142542\_contig\_number\_24669SRS142599\_contig\_number\_19495SRS142781\_contig\_number\_1885SRS142890\_contig\_number\_41714SRS143372\_contig\_number\_6498SRS143417\_contig\_number\_32250SRS143466\_contig\_number\_18050SRS144183\_contig\_number\_40455SRS144297\_contig\_number\_11929SRS144506\_contig\_number\_48800SRS144537\_contig\_number\_37740SRS144603\_contig\_number\_2474SRS147272\_contig\_number\_contig-100\_441.26007SRS147425\_contig\_number\_11221SRS148196\_contig\_number\_43779SRS148319\_contig\_number\_12556SRS148511\_contig\_number\_564SRS149075\_contig\_number\_17525SRS149244\_contig\_number\_1911SRS149325\_contig\_number\_contig-100\_335.110469SRS893187\_contig\_number\_19578SRS893231\_contig\_number\_7157SRS893253\_contig\_number\_2206SRS893341\_contig\_number\_13921SRS893342\_contig\_number\_1676SRS893358\_contig\_number\_12827SRS893366\_contig\_number\_contig-100\_693.156144SRS893378\_contig\_number\_13958SRS971276\_contig\_number\_33956
147
species
28116

1262743
species
1

SRS893382\_contig\_number\_967


SRS014613\_contig\_number\_4039SRS020394\_contig\_number\_16803SRS050801\_contig\_number\_3699SRS064276\_contig\_number\_10431SRS893383\_contig\_number\_20382
species
2292956
5


SRS148159\_contig\_number\_47119
1
species
556259

1
family
1853231

1
574697
genus

1
species
2042309

SRS018541\_contig\_number\_10022
